# Phage therapy against *Pseudomonas aeruginosa* biofilm infections: challenges and advanced strategies

**DOI:** 10.3389/fmicb.2026.1899503

**Published:** 2026-10-19

**Authors:** Qianhe Xu, Shaoping Liu, Juan Xi

**Affiliations:** 1 School of Laboratory Medicine, Hubei University of Chinese Medicine, Wuhan, Hubei, China; 2 Medical Science Research Center, Zhongnan Hospital of Wuhan University, Wuhan, Hubei, China

**Keywords:** biofilm, extracellular matrix, phage competition, phage therapy, *Pseudomonas aeruginosa*

## Abstract

Bacteriophage therapy offers a potential solution for treating multidrug-resistant *Pseudomonas aeruginosa* infections, especially those involving biofilms that reduce antibiotic effectiveness. However, successful *in vitro* results often don not lead to consistent clinical success due to various challenges. These challenges are categorized into four areas: biofilm and bacterial defenses, phage ecological limitations, host-related pharmacokinetic and pharmacodynamic issues, and manufacturing and regulatory hurdles. In biofilms, phage activity is collectively shaped by extracellular matrix composition, receptor accessibility, and biofilm spatial structure. Dense matrix components including exopolysaccharides and extracellular DNA (eDNA) form a key barrier to phage penetration, while biofilm thickness further modulates phage access to target cells. Additionally, bacterial anti-phage mechanisms and prophage resistance decrease effectiveness, particularly in genetically diverse strains. Phage cocktail effectiveness is influenced more by replication dynamics, receptor compatibility, and dosing schedule than by the number of phages. Imbalanced replication and shared receptor targets can lead to inter-phage competition, reducing biofilm clearance compared to single-phage therapy. *In vivo*, phage activity is affected by bacterial receptor expression, mucus, immune response, and biofilm gradients that foster resistant subpopulations. Phage-antibiotic combinations can improve outcomes, but their interactions vary from synergistic to antagonistic based on dosage, timing, and bacterial strain, with resistance resensitization effects being highly variable. Clinical development is hindered by non-standardized production, simplistic preclinical models, and inconsistent regulations, though Artificial intelligence and machine learning (AI/ML) could aid in screening and optimizing phage cocktails, limited by data quality. This review discusses advanced strategies to address these barriers, with a primary focus on engineered phages, sequential administration regimens, AI-driven therapeutic design, and standardized manufacturing practices. Overall, this work aims to inform the optimization of phage therapy and support its progression toward a mainstream clinical option for chronic, drug-resistant *P. aeruginosa* biofilm infections.

## 1 Introduction

*Pseudomonas aeruginosa* is a prominent opportunistic pathogen that persists in chronic infections by forming structured biofilm communities, observed in conditions such as cystic fibrosis (CF) lung disease, ventilator-associated pneumonia, chronic wounds, and device-related infections. Within these biofilms, exopolysaccharides like Pel and Psl establish dense structural scaffolds that contribute to antibiotic tolerance and subsequent treatment failure (Jennings et al., 2021). Phage therapy has re-emerged as a potential strategy against multidrug-resistant *P. aeruginosa*; however, its effectiveness in biofilm-associated infections remains inconsistent in clinical settings, as activity against planktonic bacteria does not reliably lead to the clearance of sessile, matrix-embedded cells (Billaud et al., 2025; Walton et al., 2025).

Several recent reviews have explored the application of phage therapy in the treatment of *P. aeruginosa* infections. Hwang et al. conducted a comprehensive analysis of phage strategies targeting chronic lung infections in patients with CF, emphasizing airway delivery and mucus-penetrating formulations (Hwang et al., 2026). Building upon this foundation, the current mini-review examines biofilm-specific failure mechanisms across a wider spectrum of infection contexts, including airway, device-associated, and wound biofilm models, alongside clinical evidence. This review posits biofilm formation as a primary factor contributing to the failure of phage therapy in these infections and categorizes the associated challenges into four interrelated levels: biofilm structure and bacterial defenses, phage ecology, host-related pharmacokinetic and pharmacodynamic constraints, and manufacturing and regulatory challenges. For each level, the review aligns the underlying mechanisms with the necessary measurements and levels of evidence required to assess potential solutions. Figure 1 illustrates the conceptual framework, while Table 1 outlines the experimental systems, endpoints, and evidence tiers pertinent to various study types.

**Figure 1:**
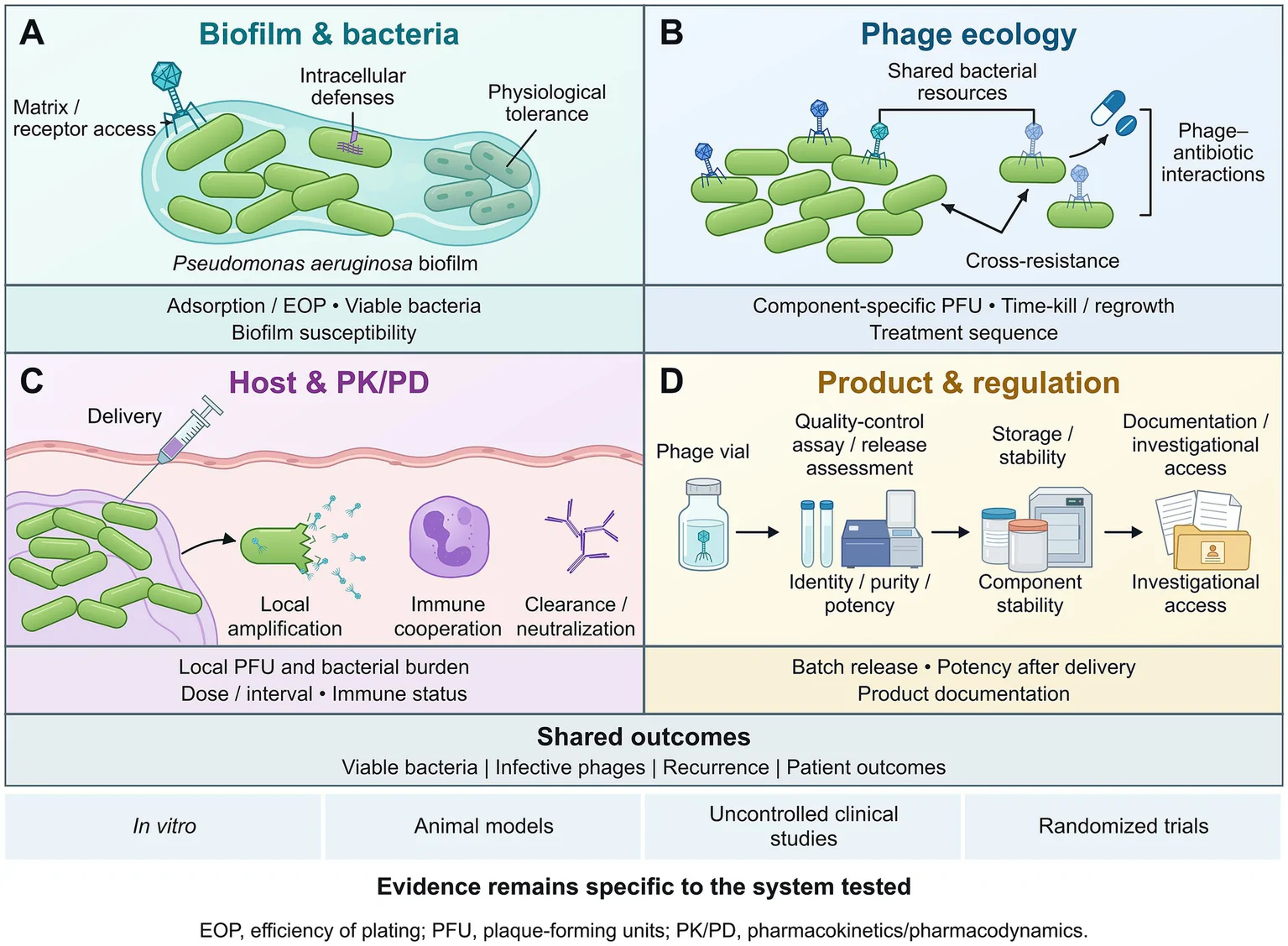
Four-level framework for evaluating phage therapy against *P. aeruginosa* biofilm infections. **(A)** Biofilm and bacterial barriers link access, receptor availability, and resistance to susceptibility measurements. **(B)** Phage ecology links cocktail compatibility and treatment sequence to component-specific phage recovery and bacterial regrowth. **(C)** Host-related constraints connect PK/PD and immunity to local exposure and bacterial burden. **(D)** Manufacturing and regulation connect product quality to potency after delivery and product documentation. Cross-resistance and the illustrated interactions are context-dependent. Shared outcomes and evidence settings guide interpretation; the diagram does not quantify effects or establish a hierarchy of clinical effectiveness. EOP, efficiency of plating; PFU, plaque-forming units; PK/PD, pharmacokinetics/pharmacodynamics. Representative sources: Bürkle et al. (2025), Cha et al. (2025), Chaudhry et al. (2017), European Medicines Agency (2025), Roach et al. (2017), and Walton et al. (2025). Figure drawn by the authors.

**Table 1:** Selected evidence relevant to *P. aeruginosa* biofilm therapy.

Study	Evidence setting	Intervention/comparison	Readout/finding	Inference limit
Walton et al. (2025)	*In vitro* biofilm model	Clew-1; receptor and biofilm assays	Psl-dependent infection	Receptor finding; clinical clearance untested
Luo et al. (2026)	In vitro engineering	Enzyme-expressing PaGZ-1 vs. wild type	Both inhibit formation; only lactonase variant improves mature-biofilm biomass reduction vs. wild type	Enzyme-specific proof of concept
Bürkle et al. (2025)	Planktonic, cell and biofilm models	JG005/JG024; sequence; meropenem	Replication and viable-cell responses differ	Improved replication need not improve killing
Namonyo et al. (2023)	In vitro clinical-isolate biofilms	Individual phages vs. cocktails	Biomass and living-cell endpoints	Model-specific effects; no clinical extrapolation
Chaudhry et al. (2017)	In vitro plastic/epithelial biofilms	Phages with different antibiotics and timing	Drug- and schedule-dependent effects	Not a clinical dosing rule
Kim et al. (2024)	Isolate screening; mouse wound model	Phage cocktails in mice; antibiotic combinations *in vitro*	Preclinical antibacterial activity	Wound evidence does not establish airway efficacy
Roach et al. (2017)	Acute mouse pneumonia	Phage treatment with altered immune status	Neutrophil-dependent infection resolution	Chronic biofilms not directly tested
Chan et al. (2025)	Uncontrolled CF clinical report	Personalized inhaled phages	Microbiological/functional associations	No randomized causal comparison
Weiner et al. (2025)	Randomized trial, Part 1; n = 9	BX004-A vs. placebo; background antibiotics	Safety, delivery; exploratory bacterial burden	Small cohort; no proof of synergy
Jault et al. (2019)	Randomized burn-infection trial	Topical cocktail vs. standard care	Slower bacterial reduction with tested phage regimen	Product titer loss complicates interpretation
Bayat et al. (2026)	In vitro; mouse implant infection	ML-selected pair plus composite hydrogel	Integrated platform activity	AI and delivery effects not independently established

Findings and limitations are study-specific; clinical infection outcomes do not directly measure biofilm eradication. CF, cystic fibrosis; ML, machine learning.

## 2 Biofilm and bacterial barriers

### 2.1 Matrix access, receptors, and physiological tolerance

The biofilm matrix in *P. aeruginosa* acts as a barrier and interaction site for phages. It consists of exopolysaccharides (Pel, Psl, alginate), extracellular DNA (eDNA), proteins, and lipids, forming a complex structure that can impede treatments, as seen in CF sputum (Jennings et al., 2021). Pel stabilizes the matrix, alginate increases viscosity, eDNA creates cross-links, and charged components can trap phages and antimicrobials, limiting phage penetration. However, the matrix can also serve as a ligand; for example, Psl is the receptor for the phage Clew-1, and Psl-dependent phages like Bruynoghevirus LUZ24 are more active in minimal media due to changes in surface Psl levels (Walton et al., 2025; Billaud et al., 2025). Removing matrix material does not always improve phage effectiveness; it might lower infection rates by eliminating essential receptors, despite better diffusion. Therefore, matrix composition, receptor availability, and growth conditions are crucial.

Quorum sensing (QS) influences the matrix-receptor interaction affecting phage adsorption. In the BUCT640–PAO1 system, deleting the lasI gene increased phage adsorption, while adding an external las signal restored normal conditions. Deleting the rhlI gene had no such effect, suggesting las signaling reduces adsorption by affecting biofilm differentiation and thickness (Cao et al., 2025). Since BUCT640 uses Psl as a receptor, Cao et al. proposed that thicker biofilms hinder phage access. A thick extracellular matrix and high cell densities may also slow phage diffusion, though they could not link the phenotype to consistent transcriptional regulation of Psl synthesis.

It is crucial to distinguish between adsorption failure and post-entry failure, as receptor availability varies with growth conditions, biofilm state, and quorum-sensing-regulated matrix composition (Billaud et al., 2025; Cao et al., 2025). Even with successful adsorption, intracellular defenses may prevent productive infection, beyond the matrix–receptor interface scope. In biofilms, nutrient and oxygen gradients support both dormant and active cells, which respond differently to phages. This physiological tolerance reflects a cell’s metabolic state and can change with better growth conditions, unlike heritable resistance, which persists after regrowth. Accurate measurement requires separate reporting of metrics like matrix biomass, viable bacteria, dispersed cells, and infective phage recovery, as reduced biomass does not always mean bacterial killing (Namonyo et al., 2023).

### 2.2 Host range, intracellular defenses, and acquired resistance

Host range describes which bacterial strains support a defined phage activity under stated conditions. Adsorption assays test phage attachment; efficiency of plating compares plaque formation on a test strain with the reference host; and plaque and time-kill assays assess productive infection and population suppression. Because clearing spots can occur without productive infection, spot tests can overestimate the productive host range relative to efficiency of plating, as demonstrated in *Escherichia coli* (Khan Mirzaei and Nilsson, 2015). Strain diversity in chronic infections limits effectiveness. When a six-phage cocktail was tested against *P. aeruginosa* isolates from chronic lung infections, efficacy varied markedly between strains and was predicted by bacteria–phage infection-network structure and the number of defense systems per genome; Czernuszka et al. concluded that fixed, off-the-shelf products are unlikely to be broadly effective against chronic respiratory isolates (Czernuszka et al., 2025). Screening conditions can also discard useful phages. Clew-1 lysed efficiently in liquid culture and in biofilms yet failed to plaque on wild-type PAO1 in conventional top-agar assays, because plaque formation required a sufficiently large Psl-expressing subpopulation, and Psl expression depends on the c-di-GMP signaling that accompanies surface growth (Walton et al., 2025). The medium-dependent Psl phenotypes reported for LUZ24 point the same way (Billaud et al., 2025), which is why susceptibility results should always state the screening conditions on which they rest.

Once a phage has adsorbed to a host cell, infection can still fail through intracellular defense mechanisms, which are distinct from receptor loss or masking. The significance of these defenses varies among phages. In isolates from chronic lung infections, cocktail performance was predicted by the structure of the infection network and the genomic repertoire of defense systems (Czernuszka et al., 2025). In a collection of 75 isolates from chronic CF infections, each challenged with four lytic phages, 216 of 300 interactions (72%) were classified as resistant when spot tests were combined with broth infection curves (Blasco et al., 2025). The isolates carried five to nineteen defense systems apiece; resistance correlated weakly but significantly with system number, while the presence or absence of a matching receptor remained an independent determinant (Blasco et al., 2025). Prophages add complexity, but superinfection exclusion is separate from the structural changes they cause in biofilms. Isolates with Pf were significantly more likely to resist challenge phages, with Pf genomes encoding defenses at various stages, such as PfsE blocking type IV pilus extension (Blasco et al., 2025). Pf carriage, however, is an association, not a proven mechanism, and does not guarantee superinfection exclusion or validate Pf-targeting drugs on its own.

Resistance emerging during treatment requires longitudinal phenotypic testing rather than a single baseline determination. Receptor changes, such as those affecting LPS O-antigen or Psl, may confer cross-resistance across phages, but some alterations instead increase susceptibility to another phage (Billaud et al., 2025; Bürkle et al., 2025); initial host coverage and resistance suppression are therefore separate cocktail-selection objectives. Functional testing should follow the bacteria over time: we recommend sampling multiple colony morphotypes, retesting survivors, and using genomic findings to support rather than replace phenotypic assays (Czernuszka et al., 2025).

### 2.3 Matrix-targeted engineering: evidence and limits

A more direct approach is to equip phages with matrix-active enzymes. Luo et al. used CRISPR-Cas9 to modify the genome of the broad-host-range Pakpunavirus PaGZ-1, creating variants that expressed either the biofilm-modulating enzyme AiiA or a phage-derived depolymerase (Luo et al., 2026). These enzymes, not the genome editing, provided matrix-targeting activity, while the phage’s structure and replication remained unchanged. Both modified phages effectively prevented new biofilm formation, but only the AiiA-expressing variant reduced biomass in 36-h-old biofilms compared to the wild-type. Thus, preventing biofilm formation and disrupting existing ones are different outcomes, and crystal violet staining alone cannot distinguish between true bacterial killing and matrix degradation.

Receptor biology and phage engineering findings indicate that enzyme selection for biofilm-targeted phages must account for both substrate specificity and potential impacts on phage adsorption. For phages that use matrix components as adsorption receptors, expressing enzymes that degrade those same components could reduce attachment efficiency and blunt overall lytic activity (Billaud et al., 2025; Luo et al., 2026; Walton et al., 2025). Activity against mature, highly cross-linked biofilms and compatibility with intended clinical delivery routes require independent evaluation. These *in vitro* engineering studies offer proof of concept and warrant further investigation, but do not establish clinical efficacy in humans.

## 3 Phage ecology and combination therapy

### 3.1 Cocktail compatibility and treatment sequence

Increasing the number of phages in a cocktail can broaden bacterial strain coverage, but it also introduces competitive interactions between phages that share the same host population. Bürkle et al. observed strong genotype- and growth-state-dependent competition between phages JG005 and JG024 in *P. aeruginosa* biofilm models (Bürkle et al., 2025). Administering JG024 prior to JG005 alleviated replicative interference and maintained titers of both phages, but this did not translate to a significant increase in overall bacterial lysis. This finding highlights an important distinction: preserving replication of all component phages is not the same as improving bacterial clearance.

In an *in vitro* study using clinical *P. aeruginosa* isolates, Namonyo et al. reported 57.6–88.5% biofilm biomass reduction after 72 h at an initial multiplicity of infection (MOI) of 10 for single-phage treatments, while multi-phage cocktails performed equivalently to or worse than individual phages (Namonyo et al., 2023). These results demonstrate that cocktail performance is highly context-dependent. Receptor-complementarity screening, in which phages targeting distinct surface receptors are paired, represents an alternative design approach with experimental support (Kim et al., 2024). When evaluating candidate cocktails, each mixture should be directly compared to its individual components across multiple endpoints: host range coverage, viable cell killing, component-specific phage titers, and bacterial regrowth over time. Based on current evidence, there is no universally optimal cocktail size, nor is sequential dosing broadly superior to simultaneous administration across all infection models.

### 3.2 Phage–antibiotic combinations

Phage–antibiotic combinations are widely investigated as a strategy to enhance biofilm clearance, but their interactions are highly complex and cannot be predicted from antibiotic class alone. Combination effects depend on the specific antibiotic and concentration, bacterial physiological state, phage strain, and the timing of administration. In an *in vitro* plastic-surface biofilm model using strain PA14, Chaudhry et al. found synergy between phages and ceftazidime at both 1 × and 8 × minimum inhibitory concentration (MIC), and with ciprofloxacin at 1 × MIC. At 8 × MIC, ciprofloxacin was facilitative but below the predefined synergy threshold. Interactions with aminoglycosides varied by concentration and treatment schedule, and simultaneous colistin treatment showed no synergy in the same model system (Chaudhry et al., 2017). Several mechanisms may explain this variability: at high concentrations, antibiotics can inhibit bacterial metabolism and reduce the cellular resources necessary for phage replication, or they may disrupt intracellular phage production pathways. It is crucial to recognize that absence of synergy does not equate to antagonism. According to standard definitions, antagonism occurs when the combined treatment is less effective than the more efficacious monotherapy alone (Chaudhry et al., 2017). Consequently, broad antibiotic class labels are not reliable predictors of interaction outcomes for individual bacterial isolates.

Treatment order is a critical experimental variable. In the PA14 biofilm model, administering gentamicin or tobramycin at 8 × MIC 24 h after phage addition resulted in lower viable cell densities than simultaneous administration (Chaudhry et al., 2017). However, since measurements were taken 48 h after phage inoculation, the delayed-antibiotic groups also had shorter total antibiotic exposure, making it difficult to isolate timing effects from exposure duration. These findings do not justify delaying clinically indicated antibiotic therapy. Rigorous combination studies should compare simultaneous and staggered exposure schedules, include both individual monotherapy arms, and report viable bacterial counts and phage kinetics with a defined interaction criterion. Outperforming both monotherapies does not automatically constitute synergy under a formal independent-action model.

Phage–antibiotic combination activity has been demonstrated in systems beyond standard microplate assays. Phages paired with ceftolozane/tazobactam exhibit activity in a mature endotracheal tube biofilm model that more closely mimics ventilator-associated pneumonia conditions (Oliveira et al., 2024). Receptor-complementary phage cocktails have also shown efficacy in murine wound infection models, though the associated antibiotic combination experiments from that study were conducted only *in vitro* (Kim et al., 2024). While these more physiologically relevant models support further investigation, they do not yet establish an optimal clinical regimen for device-associated or chronic airway biofilm infections.

Evolutionary trade-offs introduce an additional layer of complexity. In experimental *P. aeruginosa* populations, selection for resistance to the OprM-targeting phage OMKO1 resulted in increased susceptibility to certain antibiotics (Chan et al., 2016). This resensitization effect depends on both the specific resistance mechanism and the antibiotic class, and cannot be assumed to apply universally to all phage-resistant mutants. For both preclinical and clinical studies, post-treatment phage susceptibility and antibiotic MICs should both be measured to capture these evolutionary dynamics. Computational models integrating phage dynamics, antibiotic activity, resistance evolution, and immune responses can generate testable predictions, but such modeling outputs are not equivalent to empirical data from animal or clinical studies (Rodriguez-Gonzalez et al., 2020).

Moving from preclinical models to human clinical evidence, findings require careful, nuanced interpretation. An uncontrolled case series of personalized inhaled phage therapy in patients with CF reported associations between treatment and improvements in both microbiological parameters and clinical function (Chan et al., 2025). In the first part of the randomized BX004-A trial, nine participants received a nebulized phage cocktail or placebo in addition to standard inhaled antibiotics. The trial was designed primarily to evaluate safety and tolerability, with microbiological outcomes included as exploratory endpoints (Weiner et al., 2025). Neither study design can confirm definitive pharmacological synergy or define an optimal antibiotic-phage dosing sequence. Small cohort sizes, concomitant background therapy, and variability across infection sites all limit causal inference and broader generalization of findings.

## 4 Host-related and PK/PD constraints

### 4.1 Exposure and bacterial amplification

Phage pharmacokinetics and pharmacodynamics (PK/PD) differ fundamentally from conventional antibiotics, as phage exposure at the infection site depends on both the administered dose and host-dependent bacterial amplification. The density-dependent kinetics central to phage therapy were first systematically formalized by Payne and Jansen, whose framework remains a cornerstone of preclinical PK/PD modeling (Payne and Jansen, 2001). Evaluating preclinical models requires accounting for multiple interrelated factors: local density of susceptible bacteria, phage adsorption kinetics, burst size and latent period, resistance emergence, and rates of phage clearance and inactivation (Cha et al., 2025; Rodriguez-Gonzalez et al., 2020). Latent period is a particularly critical parameter, though some mechanistic models represent its effects indirectly rather than modeling it explicitly (Cha et al., 2025). In basic PK/PD models, net phage amplification requires production to outpace clearance and inactivation; the resulting replication threshold is highly model-dependent and does not translate to a universal clinical bacterial count cutoff (Rodriguez-Gonzalez et al., 2020). Additional complexities include tissue compartmentalization and spatially heterogeneous phage-bacteria interactions within structured biofilms (Cha et al., 2025; Rodriguez-Gonzalez et al., 2020). While mechanistic models are valuable for organizing these dependencies, models calibrated in acute infection models require independent validation before being applied to chronic biofilm infections.

*In vitro* MOI refers to the initial ratio of phage particles to bacterial cells in a defined assay system. When translating to clinical dosing, phage exposure must be considered in the context of *in vivo* bacterial burden, accessible tissue compartments, receptor expression, and the proportion of administered phage that remains infective at the infection site. For this reason, *in vitro* MOI values do not translate directly to clinical doses. Clinical dose-finding studies should report the administered dose in infective units, delivery route, administration volume, dosing interval, formulation details, and measurable local phage exposure where possible. Mechanistic modeling by Cha et al. predicts a non-monotonic relationship between initial phage dose and subsequent total phage exposure, because rapid bacterial depletion at high doses limits further phage amplification (Cha et al., 2025). Repeated dosing strategies should be evaluated based on local phage persistence, bacterial regrowth kinetics, and local tolerability, rather than justified by a presumed universal benefit of low initial MOI.

### 4.2 Immunity and delivery

Host immunity exerts a dual effect in phage therapy, as it both facilitates bacterial clearance and modulates phage persistence at sites of infection. In an acute mouse model of *P. aeruginosa* pneumonia, Roach et al. showed that neutrophils are critical for effective phage-driven infection resolution, including control of phage-resistant bacterial subpopulations (Roach et al., 2017). This suggests that innate immunity acts synergistically with phages to clear infection, rather than serving solely as a pathway for phage clearance. These findings were derived from acute experimental pneumonia, however, and cannot be directly extrapolated to chronic biofilm infections or immunocompromised patient populations.

Immune modulation of phage exposure and immune-mediated antibacterial cooperation require separate assessment. Neutralizing antibodies that reduce phage infectivity have been detected in serum from patients receiving phage therapy for a range of infections (Łusiak-Szelachowska et al., 2014). While this points to neutralization as a potential mechanism limiting phage exposure, it does not follow that serum neutralization uniformly predicts treatment failure across all phage products, delivery routes, or patient populations.

Local delivery approaches may improve phage access to biofilm bacteria, but their efficacy depends on confirming that infective phage particles survive administration and reach the target compartment in sufficient quantities. The BX004-A trial offers direct clinical evidence that phage particles can be delivered to the airways via nebulization, along with preliminary safety data (Weiner et al., 2025). This finding, however, does not validate all aerosol formulations nor does it confirm eradication of established airway biofilms.

Beyond immune-related biological constraints, formulation and delivery design represent another key translational barrier, and require separate assessment of phage viability, tissue distribution, and retained antibacterial activity. Physicochemical stability during storage and effective infective exposure in mucus or tissues are distinct endpoints, and strong performance in one does not guarantee success in the other.

The PhagoBurn trial illustrates this translational gap. In the trial, product titer loss during storage and administration complicated result interpretation, with bacterial burden declining more slowly in the phage treatment arm than with standard care (Jault et al., 2019). This underscores that preserving infective potency across the full delivery pathway is critical to clinical efficacy.

## 5 Manufacturing, regulation, and supporting tools

### 5.1 Product quality and access pathways

Robust phage manufacturing processes rely on consistent genomic identity and reproducible functional potency, with rigorous multi-stage quality control spanning upstream production, downstream purification, and final formulation, in line with the draft guideline on phage therapy quality aspects issued by the European Medicines Agency (EMA) (European Medicines Agency, 2025). Pyrogen and endotoxin control is a core safety priority, as production uses Gram-negative bacterial hosts that release lipopolysaccharide during lysis; validated removal steps including tangential flow filtration, anion-exchange chromatography, and detergent-based extraction reduce endotoxin to clinically acceptable levels, quantified in final batches by the limulus amebocyte lysate assay. Residual host-cell DNA and proteins carry immunogenic and reactogenic risks and require strict quantitative monitoring: residual genomic DNA is measured by quantitative PCR, residual host proteins by total protein or targeted immunoassays, and purification processes are optimized to keep these contaminants below predefined regulatory thresholds. Sterility is a fundamental requirement for all therapeutic phage products, maintained through aseptic processing across harvest, purification, and fill–finish operations. Sterile filtration acts as the primary bacterial clearance method, while terminal sterilization is only used where fully compatible with phage infectivity, and all final batches undergo compendial sterility testing to confirm the absence of viable bacteria and fungi. Phage particle aggregation is another key quality concern, as it reduces specific infectivity, alters biodistribution, and increases immunogenic risk; formulation parameters such as pH, ionic strength, and excipient composition are optimized to minimize aggregation, which is routinely monitored by dynamic light scattering and transmission electron microscopy. For multi-phage cocktails, component-specific potency testing is essential, as bulk titer measurements can mask declines in individual phage strains caused by differential stability or purification bias.

Regulatory classification of therapeutic phage products in the European Union cannot be reduced to a single category. According to the official EMA definition, Advanced Therapy Medicinal Products (ATMPs) comprise gene therapy, somatic cell therapy, and tissue engineering products (European Medicines Agency, n.d.). Classification of an individual phage product requires case-specific assessment against relevant legal criteria and its intended mechanism of action; genetic modification of the phage alone does not automatically trigger ATMP classification. Consequently, ATMP hospital exemption provisions do not serve as a generic access pathway for all phage therapy products.

In the United States, investigational phage therapy falls under the standard Investigational New Drug (IND) regulatory framework. For patients with serious or life-threatening conditions who lack satisfactory alternative treatment options, access outside formal clinical trials is available through expanded access mechanisms, including emergency single-patient IND arrangements. These pathways are outlined in joint FDA–NIAID guidance, consistent with the agency’s standing expanded access regulations (US Food and Drug Administration and National Institute of Allergy and Infectious Diseases, 2021; US Food and Drug Administration, n.d.). This form of access is investigational by design and does not constitute marketing approval or a formal finding of clinical effectiveness. For personalized phage products, any modifications require documented quality control protocols and proactive regulatory engagement, and do not automatically waive standard requirements for product characterization and manufacturing oversight.

### 5.2 AI/ML as an auxiliary tool

Artificial intelligence and machine learning (AI/ML) approaches are increasingly used to prioritize phage candidates for experimental screening and refine cocktail design. Bayat et al. paired AI-driven genomic clustering with an injectable, phage-loaded hydrogel, evaluating the integrated platform in *P. aeruginosa* biofilm assays and a murine implant infection model (Bayat et al., 2026). While this work provides a promising preclinical proof of concept for AI-assisted development, it does not demonstrate that AI-selected cocktails consistently outperform conventionally designed cocktails across diverse infection settings. Additionally, the combined study design means the individual contribution of AI selection cannot be disentangled from the effects of the hydrogel delivery formulation.

While perspective articles have outlined broader potential applications of AI/ML in phage therapy development, they do not provide comparative therapeutic evidence (Doud et al., 2025). Rigorous validation of AI screening models calls for testing across independent panels of clinical isolates, experimental confirmation of predicted phage–bacteria interactions, and head-to-head comparison against a defined conventional screening baseline with matched experimental effort. Such evaluations should also partition closely related strains between training and test sets to mitigate the risk of data leakage and overinflated performance estimates. Taken together, AI/ML supports candidate selection and optimization within the four-level translational framework, but it is not a substitute for phenotypic susceptibility testing, product quality control, or clinical trial evaluation (Table 1).

## 6 Discussion

A core challenge in translating phage therapy to biofilm-associated infections is aligning well-characterized mechanistic activity with appropriate, clinically meaningful endpoints. These endpoints span receptor binding affinity, biofilm biomass reduction, sustained replication of cocktail components, reductions in viable bacterial counts, and improved patient outcomes, each addressing distinct scientific and clinical questions. The four-level framework outlined in this review maps these endpoints to their underlying mechanisms and corresponding evidence contexts, allowing for cross-study comparisons without presupposing a single universal bottleneck to efficacy.

This analysis identifies three key practical research priorities for advancing phage therapy. First, preclinical evaluation of phage candidates should use clinically relevant bacterial isolates and fully mature preformed biofilms, include component-specific phage recovery assays, and assess bacterial regrowth over extended timeframes. Second, studies of phage–antibiotic combinations should systematically evaluate antibiotic concentration, treatment sequencing, and resistance-associated evolutionary trade-offs, rather than relying on *a priori* assumptions of synergy. Third, clinical trials should explicitly link delivered product potency and local phage exposure to key outcomes, including microbiological response, symptom control, infection recurrence, and safety endpoints. More physiologically relevant preclinical models can accelerate this translational progress, but only after their predictive value for clinical outcomes has been rigorously validated.

This review has several limitations. Given its focused scope, it does not attempt to comprehensively cover all published studies, and heterogeneity in study designs and endpoints across the literature prevents direct quantitative comparison. Most mechanistic evidence comes from laboratory reference strains tested under controlled *in vitro* conditions, while acute animal infection models only partially capture the complexity of chronic biofilm disease in humans. Uncontrolled clinical observations and early-phase trials support further exploration, but they do not confirm durable biofilm eradication or a broadly applicable dosing strategy (Chan et al., 2025; Jault et al., 2019; Weiner et al., 2025). Phage therapy remains a promising adjunct antimicrobial approach, but its clinical benefit must be robustly demonstrated for well-defined products, specific patient populations, and targeted clinical indications.
